# Letter to the Editor: On the Critical Nature of Psychosomatics in Clinical Practice

**DOI:** 10.32872/cpe.16309

**Published:** 2025-08-29

**Authors:** Emanuele Maria Merlo, Liam A. M. Myles, Gabriella Martino

**Affiliations:** 1 Department of Biomedical and Dental Sciences and Morphofunctional Imaging, University of Messina, Messina, Italy; 2 Department of Experimental Psychology, University of Oxford, Oxford, United Kingdom; 3 Department of Clinical and Experimental Medicine, University of Messina, Messina, Italy

## Abstract

- Scientific dialogue on current trends referred to psychosomatics results necessary.
- Fostering dialogue and dissemination represents scientific advancement.
- Clinical psychology deepening psychosomatics provides multifactorial results.

Dear Editors,

Acknowledging this journal's substantial commitment to advancing diverse applications in clinical psychology, this contribution aspires to cultivate a scientific dialogue within the psychosomatic domain.

Building upon this journal’s publication concerning the work of Kleinstäuber et al. (2024), which highlighted the paucity of systematic contributions on comprehensive approaches and biopsychosocial models to inform the identification of aetiological factors, fostering dialogue and dissemination remains of paramount importance.

The current trends in clinical psychology seem to recognize the need for a multidisciplinary approach and increased communication (Kleinstäuber et al., 2023). These areas of interest have been addressed by the journal, thereby inspiring opportunities for initiating an innovative and international scientific discourse. The works of Fischer and Ehlert (2019), Frostholm and Rask (2019), as well as Weigel et al. (2022), have demonstrated considerable sensitivity to topics such as diagnosis, intervention, aetiological factors, and multidisciplinary approaches.

Differentiation between organic and endogenous causes, as well as psychological and ecological factors, is frequently observed. While these distinctions may still hold necessity, epistemological considerations remind us of their unitary status. Despite this, there remains a prevalent and persistent inclination to divide domains, thereby overlooking inherently inseparable unitary realities. Systems of thought continue to exist in which the notion of a dominant metaphysics over physics remains a common inclination.

A compelling illustration of this trend is evident in the psychosomatic field, particularly regarding genuine psychosomatic and chronic conditions. The concept of psychological factors exerting influence on biological systems is traditionally regarded as accurate within the classical framework of psychosomatic theory.

Nonetheless, this conceptual accuracy may still align with a system of thought that historically regarded psychological factors as extrinsic to the physical dynamics of a suffering body, perceiving them as mere epiphenomena. Epistemologically sustainable reconfigurations are warranted, as exemplified by contemporary neuroscientific integrative approaches.

This contribution seeks to underscore the fundamental importance of fostering a debate on these prevailing trends. It aims to establish a platform for dialogue through which processes may be refined to enhance the precision and relevance of daily scientific practice.

In conclusion, insights and exchange between clinical contexts belonging to different realities produces scientific advancement that emerges necessary.
